# Dynamic hyperinflation occurs in healthy subjects during maximal voluntary ventilation

**DOI:** 10.3389/fmed.2025.1657081

**Published:** 2025-11-28

**Authors:** Audrey Herpeux, Nathalie Y. Pauwen, Roger Sergysels, Anneleen Peeters, Vincent Ninane, Marie Bruyneel

**Affiliations:** 1 Department of Pulmonology, Centre Hospitalier Universitaire Saint-Pierre, Brussels, Belgium; 2 Research Unit in Cardio-Respiratory Physiology, Exercise & Nutrition, Faculty of Human Movement Sciences, Université libre de Bruxelles, Brussels, Belgium

**Keywords:** maximal voluntary ventilation, dynamic hyperinflation (DH), operating lung volumes, inspiratory capacity (IC), end expiratory lung volume (EELV)

## Abstract

**Background:**

Dynamic hyperinflation (DH) commonly occurs during maximum voluntary ventilation (MVV) in obstructive lung diseases and is usually attributed to expiratory flow limitation. However, in healthy individuals, maximal effort typically remains insufficient to induce DH. This study aimed to determine whether DH can also develop during MVV in normal subjects without expiratory flow limitation, challenging the current concepts of its occurrence in physiological conditions.

**Methods:**

Forty-three healthy subjects (25 males/18 females; 40–68 yrs; FEV_1_: 3.4 ± 0.8 L) performed MVV at increasing breathing frequencies (ranging from 10 to 100 breaths.min^−1^) and maximal tidal volumes. Inspiratory capacity (IC) was measured at rest and every 12 s during MVV to detect DH. When DH occurred at a certain frequency (BF_DH_), a single additional MVV at a higher frequency was performed to confirm its occurrence.

**Results:**

DH occurred at or below 100 breaths.min^−1^ in 39 of 43 healthy subjects (91%), with the mean onset at 52 breaths.min^−1^. There was no significant correlation between the frequency of breathing at the onset of DH and age, body mass index, lung volumes, or flows. However, BF_DH_ was significantly and inversely correlated with resting [IC/FVC], meaning that individuals operating at lower resting ventilatory levels reached DH at lower breath frequency (BF).

**Conclusion:**

DH can occur at low and variable breathing frequencies during MVV in healthy subjects. The onset of DH may be related to the mechanical thoracopulmonary state at rest, suggesting that the baseline lung mechanics may determine the susceptibility to DH during high-intensity ventilatory maneuvers.

## Introduction

Maximum voluntary ventilation (MVV - L/min) is the maximum volume of air that can be inhaled and exhaled in 1 min. It is achieved by stimulating the subject to ventilate at the highest possible breath frequency (BF breaths.min^−1^) and tidal volume (V_T_ - L) for 12 s, and this result is extrapolated to 1 min (1).

MVV is a proxy for the maximum mechanical ventilatory capacity of the subject’s thoraco-pulmonary system, to which the ventilation observed at maximal exercise (VE_peak_) can be compared to assess respiratory reserve or to assess ventilatory limitation during exercise (2). To explore MVV, several BF have been proposed by several authors, but in 2005, based on the reports by Bernstein et al. and Miller et al., the ATS/ERS recommendations on MVV suggest using a 90 < BF < 110 breaths.min^−1^ (1, 3, 4).

In most pulmonology departments, MVV is routinely measured before starting cardiopulmonary exercise testing (CPET). However, as the measurement of MVV is highly dependent on the motivation of the patient and already exhausts the patient before exercise, a 35-fold increase in the forced expiratory volume in 1 s [FEV_1_*35] is currently used as the predicted theoretical value of the subject’s maximal ventilation (5).

Secondly, in some disease conditions, a decrease in inspiratory capacity (IC), the maximum volume of air that can be inhaled after exhalation, can be encountered while performing a CPET or even while performing an MVV or a metronome-paced incremental hyperventilation (6, 7). When IC at maximal effort (IC_max_) is decreased by at least 150 mL compared to the resting IC (IC_rest_), and the end-expiratory lung volume (EELV) increases in the same proportion, a dynamic hyperinflation (DH) is considered to occur (8, 9). It is well established that the onset of DH is often related to dynamic constraints on the thoracopulmonary system and would reflect the inability of the respiratory system to achieve true relaxation volume despite the activity of the expiratory muscles, leading to a respiratory limitation (8, 10, 11).

In obstructive conditions, the expiratory flow limitation is related to a progressive accumulation of air in the lungs over the course of breathing cycles and results in dyspnea, which considerably limits functional capacity (10).

Conversely, when healthy subjects are performing a CPET, they increase their ventilation to a maximal observed (VE_peak_), but not to the maximum predicted value (VE_pred_), and the EELV decreases below functional residual capacity (FRC), while the IC increases proportionally during exercise (6). As a result, in non-pathological situations, there should be no DH revealed during a CPET in subjects who do not reach their theoretical VE_pred_. However, it could be assumed that a DH may occur while performing an MVV; when the healthy subject ventilates above the VE_peak_, we should have observed it during a CPET.

If a DH occurs, it would be of interest to acknowledge whether DH would depend on the BF at which the MVV is achieved and on two other parameters (all interdependent): the ventilatory ratio estimated by [VE/(FEV_1_*35)] and the volume ratio estimated by [Tidal Volume /Forced Vital Capacity: V_T_/FVC].

The aims of the present study are (1) to estimate whether DH is observed in healthy subjects during a performance of MVV at incremental breath frequencies, and (2) to explore potential variables implicated in the appearance of DH.

## Materials and methods

This prospective study was conducted at the St-Pierre University Hospital in Brussels (CHU St Pierre, Brussels, Belgium). The study protocol was approved by the ethics committee of Saint-Pierre University Hospital (B076201318918). All procedures performed in studies involving human participants were in accordance with the ethical standards of the institutional and/or national research committee and with the Helsinki Declaration and its later amendments or comparable ethical standards. All included subjects provided written informed consent to participate in the study.

### Subjects

Healthy volunteers >18 Yrs old, non-smokers, with no known respiratory disease and no respiratory symptoms (e.g., dyspnea, cough) were recruited among hospital staff and their relatives. All had normal spirometry, with FEV_1_/FVC > 0.70 and FEV_1_ and FVC within the expected ranges for age, sex, and height (Table 1).

**Table 1:** Demographic and spirometric characteristics of the sample.

Demographic - spirometric parameters	All population (*n* = 43)	Gr 1 [40–49 yrs] (*n* = 15)	Gr 2 [50–59 yrs] (*n* = 16)	Gr 3 [60–69 yrs] (*n* = 12)	*p*-value
Age - Yrs	53.9 ± 7.8	44.9 ± 3.2	55.8 ± 2.5	62.8 ± 2.8	<0.001^ɸ^
Sex- F/M, *n* (%)	18 *(42%)*/25 *(58%)*	7 *(16%)*/8 *(19%)*	8 *(19%)*/8 *(19%)*	3 *(7%)*/9 *(21%)*	0.430^†^
BMI – Kg.m^−2^	24.6 ± 3.4	24.0 ± 3.5	25.3 ± 3.5	24.3 ± 3.3	0.538^ɸ^
FEV_1_ - L	3.36 ± 0.79	3.63 ± 0.93	3.21 ± 0.58	3.23 ± 0.82	0.268^ɸ^
FEV_1_-pred - %	106.2 ± 12.0	107.3 ± 12.1	107.5 ± 9.8	103.2 ± 14.7	0.595^ɸ^
FVC - L	4.31 ± 0.91	4.57 ± 1.03	4.18 ± 0.74	4.15 ± 0.97	0.395^ɸ^
FEV_1_/FVC - %	77.9 ± 4.2	79.1 ± 3.8	76.9 ± 3.9	77.6 ± 4.9	0.322^ɸ^
IC_rest_ - L	2.97 ± 0.70	3.08 ± 0.80	2.96 ± 0.60	2.84 ± 0.74	0.675^ɸ^

BMI, Body Mass Index (Kg.m^−2^); FEV_1_, Forced Expiratory Volume in 1 s (L); FEV_1_-pred, Forced Expiratory Volume in 1 s, percent of predicted value (%); FVC, Forced Vital Capacity (L); FEV_1_/FVC, Ratio of Forced Expiratory Volume in 1 s to Forced Vital Capacity (%); IC_rest_, Inspiratory Capacity at rest (L). *p*-values are derived from Chi-square tests (^†^) and from one-way Anova (^ɸ^). Italic values indicate the percentage distribution of males and females.

### Procedures

To exclude possible obstructive subjects, each participant first performed forced spirometry measurements (*M. E. C. PFT systems pocket spirometry, meeting ATS standards and calibrated daily for BTPS conditions*).

After demonstrating normal spirometry defined by FEV_1_/FVC > 0.7, we asked the subjects to breathe at a quiet, natural rate and to perform a resting inspiratory capacity procedure (IC_rest_) at least 4 times. Only the highest volume was chosen from the reproducible IC_rest_ performed. Finally, with clear instructions and active encouragement from our physiotherapist, the subjects started MVV by breathing in and out as deeply as possible relative to their FVC, with controlled BF, inhaling normal air with a low-resistance mouthpiece (*MicroGard II filter, inspiratory/expiratory resistance: <0.04* kPa/(L/s) at 1 L/s; <0.4 cmH2O/(L/s) at 1 L/s).

BF was controlled using a metronome, starting at a target BF of 10 breaths·min^−1^, which was steadily increased in steps of 10 breaths.min^−1^, until a maximum BF of 100 breaths.min^−1^ was reached. Each maneuver was separated by a rest period based on each individual’s need to recover from the previous BF session. If the target BF could not be achieved during the MVV, the maneuver was repeated, with no more than two attempts allowed. Each frequency was maintained for 12 s during MVV, with, again, an IC maneuver at the end of the MVV to detect any DH.

We considered that DH appeared for a given BF when the IC observed was reduced by at least 150 mL compared with the IC_rest_ (6, 9, 10). The ΔIC ≥ 150 mL cutoff was selected for DH because it exceeds short-term IC variability observed under supervised conditions and has been used to denote clinically meaningful DH; in healthy individuals, this threshold is conservative (12). To minimize false positives from a single IC maneuver, DH required confirmation at the next BF step (BF + 10), thereby establishing a sustained IC decline consistent with best practices for serial IC as a surrogate of EELV (13).

When DH was first observed at a given frequency (BF_DH_), it was confirmed by an additional MVV test performed at the BF_DH_ incremented by 10 breaths.min^−1^ (BF_DH_ + 10 breaths.min^−1^) to validate the recurrence of DH. In this situation, we considered that DH was observed at BF_DH_ and was documented.

As long as no DH was detected, the MVV procedure was performed with 10 breaths.min^−1^ increments in BF continue until BF_DH_ is observed or until a maximum of 100 breaths.min^−1^-BF is reached.

For each BF investigated, mean tidal volume (V_T_) was assessed based on MVV and BF performed (MVV/BF).

### Statistical analysis

To determine the BF at which more than 50% of healthy subjects experienced DH, a time-to-event (Kaplan–Meier) analysis was performed with the occurrence of DH as the dependent variable and BF or ventilatory ratio [MVV/(FEV_1_*35)] or volume ratio [V_T_/FVC] as the independent variables. The event was defined as the first IC decrease ≥150 mL *vs.* the rest, confirmed at the subsequent BF step (BF + 10), ensuring a sustained IC decline as a surrogate of EELV increase. Participants who did not develop DH by BF = 100 breaths.min^−1^ were treated as right-censored at their last observed BF. Kaplan–Meier estimates and 95% CIs were computed; between-age subgroup curves (40–49, 50–59, 60–69 yrs) were compared with the log-rank test (Mantel-Cox), and the same procedure was applied to compare curves between women and men. Censoring was assumed to be non-informative.

Plots of time-to-event analysis are displayed for (1) BF, (2) ventilatory ratio, and (3) the complement [1-(V_T_/FVC)], since V_T_/FVC decreases with BF, its complement provides an increasing variable consistent with Kaplan–Meier analysis. Participants without DH observed by BF = 100 breaths.min^−1^ were right-censored at that time point on each scale.

In order to explore potential variables to consider in the appearance of DH, correlations were estimated between BF_DH_, ventilatory ratio, and volume ratio with demographic or spirometry parameters.

The analysis performed was two-sided with *α* = 0.05, using *IBM SPSS Statistics 20*.

## Results

Between December 2013 and April 2014, 43 healthy subjects were included (18 women, 42%), aged 40–69 Yrs (mean 54 ± 8 Yrs, range 40–68 Yrs) with a mean BMI of 25 ± 3 Kg.m^−2^ (range 18–33 Kg.m^−2^), with spirometry parameters within the expected values. When we compared gender distribution, BMI, and all spirometric parameters, we did not observe any differences between the three age groups (all *p* ≥ 0.268) (Table 1).

Amongst the whole sample of 43 subjects and for the whole range of BF tested (from 10 breaths.min^−1^ to a maximum of 100 breaths.min^−1^), DH was experienced by 39 participants (91% of the sample) and was not seen in 4 participants at a maximal BF of 100 breaths.min^−1^ tested.

### Dynamic hyperinflation with respect to breathing frequency

**Figure 1:**
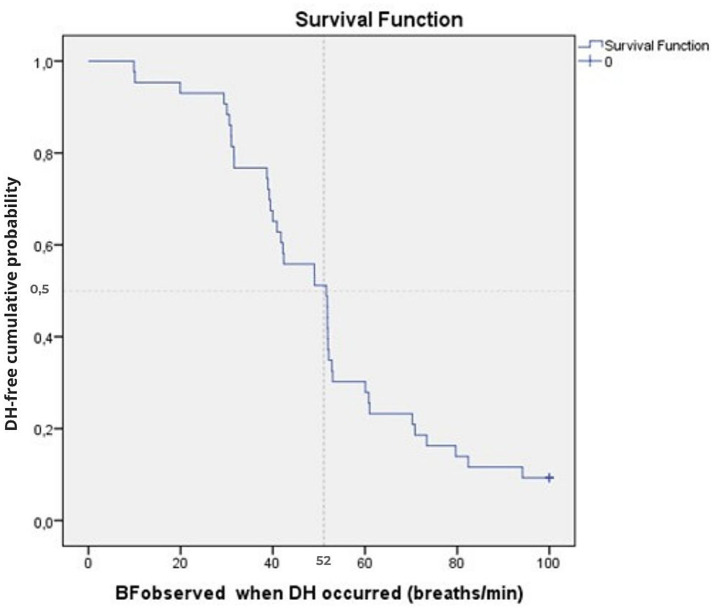
Cumulative time-to-event plots of the DH event as a function of BF for the overall sample (*n* = 43). The Kaplan–Meier curve shows the cumulative probability of remaining DH-free according to BF at which DH occurred (BF_DH_). The vertical dotted line indicates the median threshold (52 breaths.min^−1^). Tick marks indicate censored observations (4 participants censored at BF = 100 breaths.min^−1^).

The earliest onset of DH was observed at a BF of 10/min in 2 subjects (5% of the sample), and 4 of the 43 participants were right-censored at BF = 100 breaths.min^−1^ (Figure 1). Time-to-event analysis revealed that, for the overall sample, DH was found in 50% of the sample at a BF_DH_ of 52 breaths.min^−1^ [95%CI: 39–64 breaths.min^−1^]. After excluding the four subjects without DH, the mean BFDH for the 39 subjects with DH was 52 breaths.min^−1^ (Figure 1). The log-rank test showed no difference in median time-to-event BF_DH_ between the three age groups (*p* = 0.515) (Table 2), nor with gender (*p* = 0.803).

**Table 2:** Estimates of the median breath frequency (BF) of DH occurrence, for the overall sample and by age groups (*n* = 43).

Sample/subgroup	BF_DH_ (breaths.min^−1^) Median [95% CI]	*p*-value
All Sample (*n* = 43)	52 [39;64]	0.803
Women (*n* = 18)	49 [28;70]	
Men (*n* = 25)	52 [39;64]	
Gr 1 [40–49 Yrs] (*n* = 15)	52 [51;53]	0.515
Gr 2 [50–59 Yrs] (*n* = 16)	40 [35;45]	
Gr 3 [60–69 Yrs] (*n* = 12)	42 [24;61]	

BF_DH_, Breath frequency at which DH occurs (breaths.min^−1^). *p*-values are derived from the log-rank (Mantel Cox) analysis.

### Dynamic hyperinflation with respect to ventilation

The earliest occurrence of DH was noted in the first subject at a ventilatory ratio of 27% of the maximal ventilation estimated by the ratio [MVV/(FEV_1_*35)].

**Figure 2:**
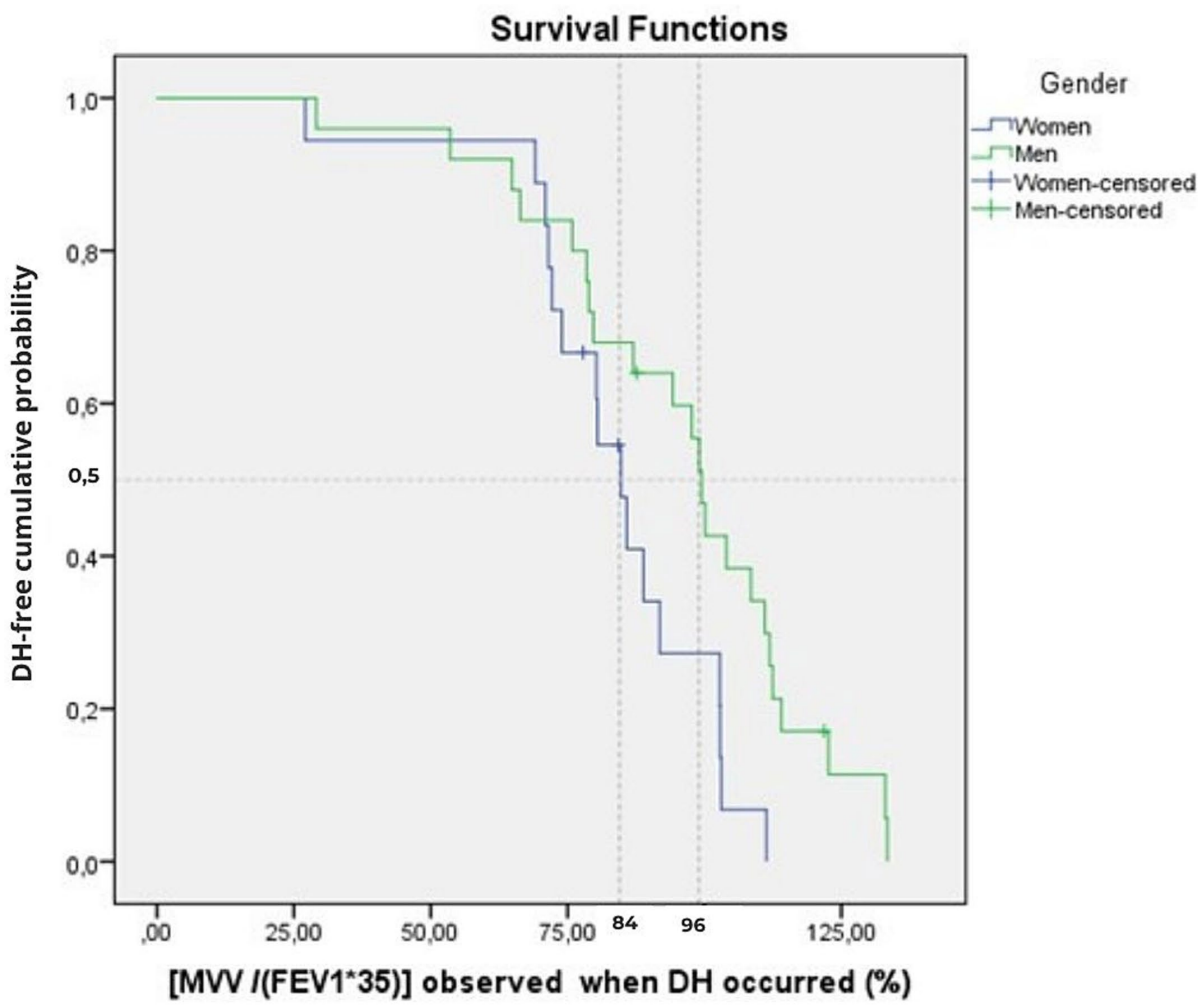
Cumulative time-to-event plots of the DH event as a function of ventilatory ratio [MVV/(FEV_1_*35)] for the women and men groups of the overall sample (*n* = 43). The Kaplan–Meier curve shows the cumulative probability of remaining DH-free according to the ventilatory ratio at which DH occurred. The vertical dotted line indicates the median threshold for women (84%) and men (96%). Tick marks indicate censored observations (four participants censored at BF = 100 breaths.min^−1^).

Time-to-event analysis revealed that, across the whole sample, DH occurred in 50% at a ventilatory ratio of 92% [95%CI: 78–106%] (Table 3; Figure 2).

**Table 3:** Estimates of the median ventilatory ratio [MVV/(FEV_1_*35)] of DH occurrence for the overall sample and by age groups (*n* = 43).

Sample/subgroup	MVV/(FEV1*35) (%) Median [95% CI]	*p*-value
All Sample (*n* = 43)	92 [78; 106]	0.022
Women (*n* = 18)	84 [75; 93]	
Men (*n* = 25)	96 [86; 106]	
Gr 1 [40–49 yrs] (*n* = 15)	92 [68; 116]	0.626
Gr 2 [50–59 yrs] (*n* = 16)	85 [61; 109]	
Gr 3 [60–69 yrs] (*n* = 12)	87 [55; 119]	

MVV/(FEV_1_ * 35): Ratio of Maximal Voluntary Ventilation (MVV) to predicted MVV (calculated as [FEV_1_ * 35]), expressed as percent (%). FEV_1_: Forced Expiratory Volume in 1 s (L). *p*-values are derived from the Log-Rank (Mantel Cox) analysis.

The log-rank test showed no difference in median ventilatory ratio [MVV/(FEV_1_*35)] of DH occurrence between the three age groups (*p* = 0.626) (Table 3), but we found a significant difference between genders, with a DH that appears at lower values in women (*p* = 0.022).

### Dynamic hyperinflation with respect to volume

The earliest occurrence of DH was noted in the first subject at a volume ratio of 77% of the measured FVC estimated by the ratio [V_T_/FVC].

**Figure 3:**
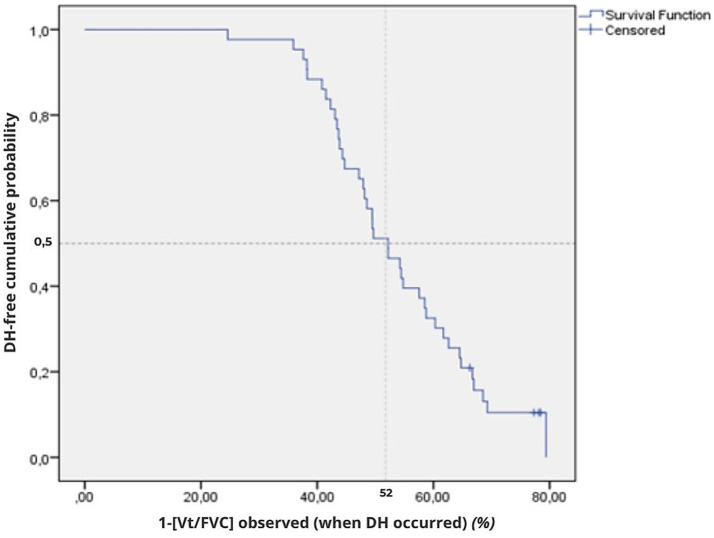
Cumulative time-to-event plots of the DH event using the complement of the volume ratio (1-[V_T_/FVC]) for the overall sample (*n* = 43). The Kaplan–Meier curve represents the cumulative probability of remaining DH-free according to [1–(V_T_/FVC)] at the onset of DH. The vertical dotted line indicates the median threshold (52%). Tick marks indicate censored observations (4 participants censored at BF = 100 breaths.min^−1^).

Time-to-event analysis revealed that, for the overall sample, DH occurred in 50% of the sample at a volume ratio reduced to 52% [95%CI: 46–58%] (Table 4; Figure 3). For the 39 subjects with DH occurrence, a median reduction of volume ratio of 52% [95%CI: 46–58%] was found (Table 4).

**Table 4:** Estimates of the median volume ratio [V_T_/FVC] of DH occurrence for the overall sample and by age groups (*n* = 43).

Sample/subgroup	V_T_/FVC (%) Median [95% CI]	*p*-value
All Sample (*n* = 43)	52 [46; 58]	0.704
Women (*n* = 18)	52 [41; 63]	
Men (*n* = 25)	50 [41; 58]	
Gr 1 [40–49 yrs] (*n* = 15)	60 [40; 80]	0.350
Gr 2 [50–59 yrs] (*n* = 16)	48 [38; 58]	
Gr 3 [60–69 yrs] (*n* = 12)	50 [49; 50]	

V_T_/FVC, Ratio of Tidal Volume (V_T_) to Forced Vital Capacity (FVC), expressed as percent (%). FEV_1_, Forced Expiratory Volume in 1 s (L). *p*-values are derived from the log-rank (Mantel Cox) analysis.

The log-rank test showed no difference in median volume ratio of DH occurrence between the three age groups (*p* = 0.350) (Table 4), nor with gender (*p* = 0.166).

### Subjects experiencing DH

**Figure 4:**
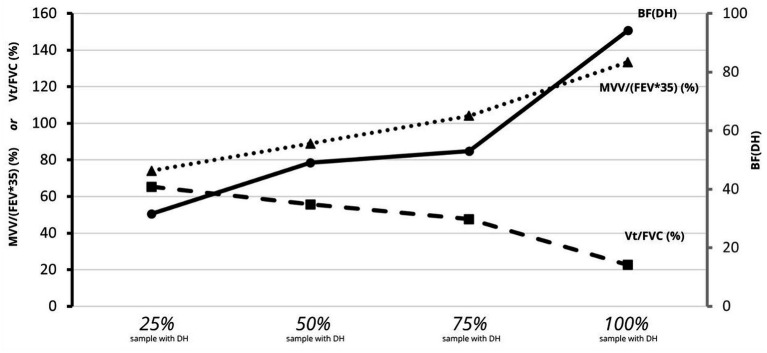
Percentile distribution for the mean of ventilatory ratio [MVV/(FEV_1_*35)], of volume ratio [V_T_/FVC], and of BF_DH_ at the onset of DH (*n* = 39). Percentile distribution for the mean of ventilatory ratio [MVV/(FEV_1_*35)], of volume ratio [V_T_/FVC], and of BF_DH_ at the onset of DH (*n* = 39). Solid black line: mean BF at DH onset; dashed line: mean [V_T_/FVC] at rest; dotted line: mean [MVV/(FEV1*35)] at DH onset.

In summary, among the 39 subjects (91% of the sample) who experienced a DH when they performed an MVV at a frequency equal to or less than 100 breaths.min^−1^, the diagram below shows that 50% of these will develop a DH (1) when the imposed BF exceeds a frequency of 52 breaths.min^−1^, (2) when MVV exceeds 92% of the maximum theoretical ventilation, and (3) when the engaged V_T_ no longer exceeds 52% of the FVC (Figure 4).

### Subjects without DH observed

Despite a BF of 100 breaths.min^−1^ being achieved, the four subjects (9% of the sample) who never experienced DH reached a maximum ventilation ratio of 87 to 131% and a minimal volume ratio between 21 and 34%.

Minimal volume ratios and maximal ventilation ratios were not observed at the precise maximal BF tested of 100 breaths.min^−1^. While minimal volume ratios were observed at a BF close to the maximum (ranged from [80–100 breaths. Min^−1^]), peak ventilation ratios appeared when BF ranged between [50–90 breaths.min^−1^].

### Comparison of subjects with and without DH

We observed that the BMI of the subjects without DH (*n* = 4) was significantly lower than the BMI of the subjects with DH (*n* = 39) (20.6 ± 2.9 *vs.* 25.0 ± 3.3 Kg.m^−2^; *p* = 0.018). When we compared other demographic or spirometric parameters, we did not observe any differences between these two groups.

### Correlations

**Figure 5:**
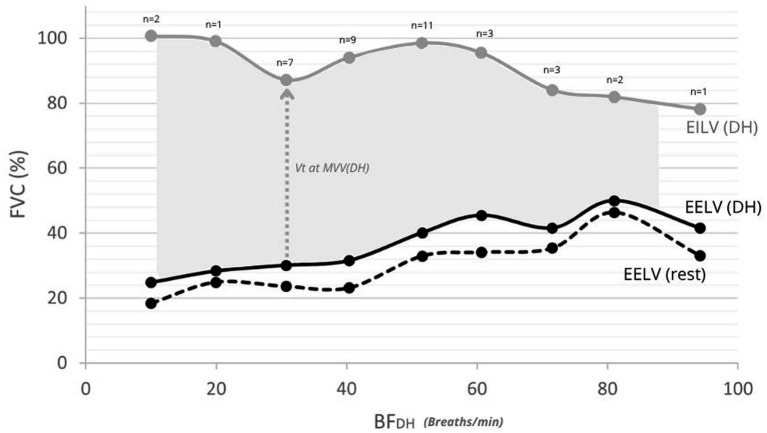
End-expiratory and end-inspiratory lung volumes (EELV/EILV) mean values at rest and at the onset of DH. Solid black line: mean EELV at DH onset; dashed black line: mean EELV at rest; grey line: mean EILV at DH onset. Numbers above the curve indicate the number of participants (*n*) contributing to each value at DH onset. The shaded area represents the mean V_T_ developed during MVV at DH onset.

Correlations revealed that the lower the resting functional residual capacity (i.e., lower resting EELV), and consequently the higher the [IC_rest_/FVC] ratio, the earlier DH appeared at lower BF (*r* = −0.824; *p* < 0.0001) and at higher relative tidal volume ratios [V_T_/FVC] (*r* = 0.847; *p* < 0.0001), as shown in Table 5 and Figure 5. Also, the higher a subject’s BMI, the more DH appears to be associated with high [V_T_/FVC] (*r* = 0.446; *p* = 0.004) (Table 5).

**Table 5:** Correlations between breath frequency of DH occurrence (BF_DH_), ventilatory ratio of DH occurrence [MVV/(FEV_1_*35)]_DH_, volume ratio of DH occurrence [V_T_/FVC]_DH,_ and demographic/spirometric variables of the subjects.

Demographic/spirometric variables	BF_DH_ R (*p*-value)	[MVV/(FEV_1_*35)]_DH_ R (*p*-value)	[V_T_/FVC]_DH_ R (*p*-value)
Age (yrs)	−0.185^®^ *(0.236)*	−0.134^®^ *(0.393)*	0.122^¥^ *(0.460)*
BMI (Kg/m^2^)	0.182^®^ *(0.268)*	0.057^®^ *(0.733)*	0.446^¥^ *(0.004)*
FEV_1_ (%)	0.022^®^ *(0.886)*	−0.038^®^ *(0.811)*	−0.068^¥^ *(0.681)*
FEF25%	0.072^®^ *(0.647)*	−0.108^®^ *(0.492)*	0.013^®^ *(0.935)*
FEF50%	0.216^®^ *(0.164)*	−0.064^®^ *(0.681)*	−0.046^¥^ *(0.782)*
FEF75%	−0.058^®^ *(0.711)*	0.124^®^ *(0.428)*	−0.148^¥^ *(0.369)*
FEF25-75%	0.171^®^ *(0.272)*	−0.006^®^ *(0.971)*	−0.028^®^ *(0.867)*
Tiffeneau (FEV_1_/FVC)	0.171^®^ *(0.273)*	0.101^®^ *(0.521)*	−0.013^¥^ *(0.936)*
ICrest	−0.317^®^ *(0.039)*	0.248^®^ *(0.109)*	0.421^¥^ *(0.008)*
ICrest/FVC	−0.824^®^ *(<0.0001)*	−0.026^®^ *(0.870)*	0.847 ^¥^ *(<0.0001)*

^¥^ Pearson’s correlation; ^®^ Spearman’s correlation. BMI, Body Mass Index (Kg.m^−2^); FEV_1_-pred, Forced Expiratory Volume in 1 s, percent of predicted value (%); FEF₂₅_%_, Forced Expiratory Flow at 25% of Forced Vital Capacity (L·s^−1^); FEF₅₀_%_, Forced Expiratory Flow at 50% of Forced Vital Capacity (L·s^−1^); FEF₇₅_%_, Forced Expiratory Flow at 75% of Forced Vital Capacity (L·s^−1^); FEF₂₅–₇₅_%_, mean Forced Expiratory Flow between 25 and 75% of Forced Vital Capacity (L·s^−1^); FEV₁/FVC (Tiffeneau index), ratio of Forced Expiratory Volume in 1 s to Forced Vital Capacity (FVC) (%); IC_rest_, Inspiratory Capacity at rest (L); IC_rest_/FVC, ratio of Inspiratory Capacity at rest to Forced Vital Capacity (FVC) (%). Values are Pearson’s correlation coefficients (r) with corresponding *p*-values. Italic values indicate *p*-values.

## Discussion

In our study, DH was observed in 91% of healthy adults during MVV, with some individuals exhibiting it at relatively low BF. DH appeared for half of the sample at the mid-range of BF 52 breaths.min^−1^ [95%CI: 39–64], in a ventilation ratio of 92% [95%CI: 78–106%], and when 52% [95%CI: 46–58%] of the volume ratio is reached.

By definition, a fall in IC at constant TLC reflects an increase in EELV, i.e., DH. The chosen ≥150 mL and our confirmation at the next BF_DH_ step (BF + 10) further limits false positives (12, 13).

However, while direct measurement of MVV (before performing a CPET) undoubtedly has the advantage of being more accurate than extrapolating maximum ventilation from [FEV_1_*35] or [FEV_1_*40], it has the disadvantage of requiring a good understanding and cooperation of the subjects to obtain, at the same time, the maximum V_T_ and the highest BF (14). To minimize underestimation of MVV, we sought to ensure consistent motivation across participants by providing standardized verbal encouragement from the same physiotherapist, who instructed each subject to generate the largest possible V_T_ during all testing sessions (14, 15).

To achieve MVV, D’Silva & Mendel recommend a BF between 70 and 90 breaths.min^−1^, while Shephard et al. propose a BF of 90–100 breaths.min^−1^ (16, 17). We observed a minimum BF of 70 breaths.min^−1^ and that 84% of our sample had already developed DH. If we consider the ATS/ERS recommendations (optimal MVV measurement to be performed at 90 < BF < 110 breaths.min^−1^), a DH is already experienced by 91% of our healthy participants at a BF of 90 breaths.min^−1^ (6).

Furthermore, Cara et al. propose to consider the optimal BF to be adopted for a meaningful MVV estimation, using the following equation: BF = [(FEV_1_*100)/FVC] (18). However, the target BF estimated by this equation does not predict DH at MVV in the healthy subjects in our study who, by definition, have an [FEV_1_/FVC] ratio within expected standards. Indeed, we observed that 92% of the subjects who developed DH did so at BF, well below the estimate from Cara’s formula. Conversely, this equation could allow for a target BF that limits the development of DH in obstructive subjects (e.g., COPD), who have a decreased [FEV_1_/FVC] ratio, by decreasing the target BF needed to achieve an MVV in these subjects.

In 1950, D’Silva & Mendel estimated that below a BF of 50 breaths.min^−1^ during an MVV, the occurrence of a DH was considered pathological (17). However, our results did not align with this consideration, as 44% of our sample of healthy subjects had already experienced a DH for BF below 50 breaths.min^−1^. A few years later, in 1957, Sadoul et al. used the term “*crenel sign*” to describe the occurrence of DH during an MVV procedure, illustrating the increase of the EELV during MVV measurement compared to the EELV observed at rest (15). While this crenelated phenomenon is characteristic of a breathing pattern typically found in obstructive subjects, Sadoul et al. also pointed out that it is not uncommon to be observed in healthy subjects at BF > 80 breaths.min^−1^. These observations in healthy subjects were later confirmed by Johnson et al. in 1999, without specifying the BF at which a DH was observable during an MVV procedure (19).

More recently, the Metronome-Paced Tachypnea (MPT) maneuver was performed at BF ≤ 40 breaths.min^−1^ for 30 s and has been developed (12, 20). Cooper et al. showed that young healthy subjects (31 ± 15 yrs) experienced DH during an MPT performed at a BF of 40 breaths.min^−1^, which we confirmed in our healthy older population (20).

### Operating-lung-volume perspective

According to our results, a very wide range of BF (with an average of 52 breaths.min^−1^) is inducing DH, even in healthy subjects, and even if they are stimulated to deploy as much volume as possible. Conversely, we observed that when the BF prescribed does not allow the healthy subject to perform more than an average of 54% of his FVC, then a DH appears.

In our cohort, during MVV, DH emerged when V_T_ was ~52% of FVC, consistent with healthy-exercise reports that V_T_ plateaus ~55–63% of vital capacity (VC) as inspiratory expansion nears thoraco-pulmonary limits: EILV approaches total lung capacity (TLC), and EELV can rise when expiratory time is shortened (13).

Extending this framework, Chuang et al. showed that V_T_/TLC discriminates healthy *vs.* obstructive/restrictive patterns better than V_T_/FVC or V_T_/IC and proposed practical V_T_/TLC bounds linked to DH *vs.* normal expansion (21). Our metronome-imposed breathing frequency during MVV shortens expiratory time, a constraint under which prior MPT work has shown EELV shifts depend on breathing pattern (20).

Although we did not measure TLC, two features support a mechanical reading: (1) by definition, a fall in IC at constant TLC denotes a rise in EELV (DH), and a ≥ 150 mL threshold with confirmation at BF + 10 limits false positives; (2) the median DH at V_T_/FVC ≈ 52% matches the expected V_T_ “plateau behavior” from Guenette et al. (12, 13).

In line with these mechanics, in our study, DH typically occurred at ~91% of predicted VE (FEV_1_*35) and a median BF ≈ 52 breaths.min^−1^, and lower resting IC_rest_/FVC predicted earlier DH, cautioning against reliance on MVV = FEV_1_*35 substitutions given their poor agreement with measured MVV (14).

This ventilatory threshold is usually well above the peak VE observed during a CPET, which is consistent with a DH rarely observed in healthy subjects at maximal effort. Moreover, we observed a significant inverse correlation between the BF_DH_ and the ratio of resting IC to FVC [IC_rest_/FVC], which represents the resting static constraints of the respiratory system (*p* < 0.0001). It reveals that the lower the FRC, the lower the BF_DH_ occurs.

### Age and gender considerations

Given the physiological loss of elastic release of the thoraco-pulmonary system with age, which affects maximal expiratory flow, we expected to observe a lower BF threshold for inducing DH in the oldest participants in our experiment. Our observations could not confirm this hypothesis since no differences were found between younger and older participants within our 40–70 Yrs age range (*p* > 0.05).

Our sample was selected from a healthy population showing expected values for the spirometry parameters, such as FEV_1_ or forced expiratory flow (FEF) at different FVC levels, that could not be related to the onset of DH, whether considering the BF, the ventilation ratio, or the volume ratio.

Conversely, we found a difference for the ventilation ratio according to gender (*p* = 0.022). This last observation could be explained by the fact that for authors, predicted maximum ventilation may vary according to gender, estimated for women at [FEV_1_*35] (what we have chosen for our entire population) and for men at [FEV_1_*38] (22).

### Fatigue considerations

Although we did not directly assess inspiratory muscle fatigue, several features of our protocol and data argue against fatigue as the primary driver of the IC fall at DH onset. Ventilation during the 12-s bouts increased from 10 to 70 breaths.min^−1^ (mean MVV: 33.6 → 98.4 L.min^−1^), showed a modest inflection at 80 (96.2 L.min^−1^), then rose again to a peak at 90 (115.3 L.min^−1^) and remained near-plateau at 100 (113.3 L.min^−1^). Among participants with a BF_DH_ of around 70–80–90 breaths.min^−1^, 60% increased from 70 → 80 breaths.min^−1^ and 60% from 80 → 90 breaths.min^−1^ and in those with BF_DH_ = 90, 33% increased from 90 → 100. These observations are consistent with performance maintenance under the imposed pacing. Together with short bout duration and individualized rest, this pattern suggests that the observed IC decrease reflects a real decrease in operating lung volume rather than a progressive decrement in ventilatory performance.

### Role of BMI

Obesity shifts operating lung volumes by reducing Expiratory Reserve Volume (ERV) and Functional Residual Capacity (FRC) while generally preserving TLC, thereby lowering resting IC and bringing EILV closer to TLC during high ventilatory demand, a mechanism that can advance DH onset (23).

Consistent with an added thoracic load, obesity blunts the ventilatory response to exercise and is associated with higher EELV from rest to peak, with EILV approaching TLC in older adults, supporting greater mechanical constraints on V_T_ expansion (24).

In our cohort, resting IC_rest_/FVC predicted the BF at DH (higher IC_rest_/FVC → earlier DH), and BMI correlated with the V_T_/FVC at DH, suggesting that higher BMI (and possibly central adiposity) may depress IC and may precipitate DH under MVV.

These observations argue for stratifying future analyses by BMI and fat distribution and for incorporating direct operating-volume measures (EELV/EILV or TLC) to quantify BMI’s modulation of DH risk.

Also, BMI, which significantly correlated with IC_rest_ (*r* = 0.333; *p* = 0.029) and may therefore play a role in CRF levels, appears to be an important parameter to consider in the onset of DH. Firstly, we observe that BMI differentiates between subjects with and without DH (*p* = 0.018), with overweight (25.0 ± 3.3 Kg.m^-2^) and standard (20.6 ± 2.9 Kg.m^−2^) BMIs, respectively. Furthermore, BMI correlates with the volume ratio at which DH appears (*p* = 0.004).

In light of these observations, it would be interesting to investigate whether, beyond obesity, being overweight and/or gender could be risk factors for DH.

### Limitations of the study

Convenience and professional-network recruitment may have introduced selection and clustering biases, combined with age and sex imbalances, further limiting generalizability.

Participants were classified as healthy based on a fixed FEV_1_/FVC > 0.70, which we acknowledge is insufficient on its own to exclude subclinical disease; ideally, static lung volumes (TLC, RV, DLCO) would be included, but these were not measured here. Nevertheless, baseline spirometry was broadly normal (FEV_1_ averaged ~106% predicted with nearly all participants ≥80% predicted), supporting a healthy classification. Despite identical instruction and encouragement from the same physiotherapist, V_T_ and therefore VE during the MVV could still have been influenced by individual motivation, while BF was the only parameter monitored.

Respiratory muscle fatigue, whether acute during repeated maneuvers or reflecting underlying strength and endurance, was not formally assessed. DH and fatigue may coexist, and serial IC (a valid proxy for EELV) cannot, by itself, exclude that some inspirations at the end of a bout were not truly maximal (13). In line with ERS perspectives that MVV is influenced by chest-wall/lung mechanics as well as “fatigue,” causal attribution from MVV alone is non-specific (14). Future studies combining IC with direct fatigue indices (e.g., MIP/MEP or SNIP, and plethysmographic EELV) could help clarify the relative contributions of these mechanisms.

The four subjects who did not develop a DH at the imposed 100 breaths.min^−1^ BF were included in the Kaplan Meier estimates, which may therefore have slightly overestimated (for the mean BF_DH_ and the mean ventilatory ratio) or underestimated (for the mean volume ratio). Future studies should specifically address the role of respiratory muscle function in relation to DH.

## Conclusion

Our study showed that when MVV is performed in healthy subjects, DH appears at very low and variable BF, which in most cases is well below the BF recommended by the ATS/ERS for optimal MVV procedure, but appears very close to the maximal predicted ventilation considered by [FEV_1_*35]. Following the guidelines, this MVV maneuver measures the efficacy of the thoraco-pulmonary system. To better approximate physiological ventilation, it would be interesting to estimate the BF at which an MVV can be obtained without DH.

The onset of DH during an MVV procedure in healthy subjects may not be a marker of their exercise breathing pattern, but rather a marker of the resting mechanics of their thoracic-pulmonary system.

The great variability of IC_rest_ conditioning the appearance of DH in healthy subjects motivates confirmatory studies with full lung volumes and inspiratory muscle testing to disentangle DH from fatigue.

## Data Availability

The datasets presented in this article are not readily available because the raw data supporting this article may be made available upon reasonable request to the corresponding author. Access to the data will require prior approval from the competent institutional ethics committee. Requests to access the datasets should be directed to audrey.herpeux@stpierre-bru.be.

## Glossary

BF (breathing frequency): Respiratory rate (breaths.min^−1^); imposed by metronome in this study
BF_DH_: First BF step at which dynamic hyperinflation (DH) is detected (IC fall ≥150 mL *vs.* rest), confirmed at BF + 10
DH (dynamic hyperinflation): Temporary rise in end-expiratory lung volume (EELV) inferred from a fall in inspiratory capacity (IC) when TLC is assumed constant
EELV (end-expiratory lung volume): Lung volume at end expiration; with constant TLC, EELV = TLC − IC
EILV (end-inspiratory lung volume): Lung volume at end inspiration; approaches TLC when V_T_ expansion is constrained
ERV (expiratory reserve volume): Volume exhaled below end-tidal expiration to RV (context term; not measured here)
FVC (forced vital capacity): Maximal volume of air exhaled forcefully after a full inspiration (to total lung capacity, TLC)
FEV_1_/FVC: Spirometric indices (forced expiratory volume in 1 s/forced vital capacity) used to document normal baseline function
IC (inspiratory capacity): Maximal inhaled volume from end expiration to TLC; primary surrogate to track changes in EELV with exercise/hyperpnea
MVV (maximal voluntary ventilation): Maximal sustainable ventilation extrapolated from a 12-s maneuver
TLC (total lung capacity): Maximal lung volume at full inflation; assumed constant over short bouts in IC-based DH assessment
V_T_ (tidal volume): Volume of a single breath; in this study, normalized as V_T_/FVC to characterize volume constraints
Ventilatory ratio [MVV/(FEV_1_*35)]: Study variable expressing measured MVV relative to a conventional estimate (used descriptively only)
Volume ratio [V_T_/FVC]: Tidal volume expressed as %FVC; used to index V_T_ “plateau” behavior at DH

## References

[1] Miller MR Hankinson J Brusasco V Burgos F Casaburi R Coates A . Standardisation of spirometry. Eur Respir J. (2005) 26:319–38. doi: 10.1183/09031936.05.00034805, PMID: 16055882

[2] Dillard TA Piantadosi S Rajagopal KR. Prediction of ventilation at maximal exercise in chronic air-flow obstruction. Am Rev Respir Dis. (1985) 132:230–5. doi: 10.1164/arrd.1985.132.2.230, PMID: 4026047

[3] Bernstein L D’Silva JL Mendel D. The effect of the rate of breathing on the maximum breathing capacity determined with a new spirometer. Thorax. (1952) 7:255–62. doi: 10.1136/thx.7.3.255, PMID: 12984412 PMC1019170

[4] Miller WF Johnson RL Wu N. Relationships between fast vital capacity and various timed expiratory capacities. J Appl Physiol. (1959) 14:157–63. doi: 10.1152/jappl.1959.14.2.157, PMID: 13641134

[5] Pride NB. Tests of forced expiration and inspiration. Clin Chest Med. (2001) 22:599–622. doi: 10.1016/S0272-5231(05)70055-1, PMID: 11787654

[6] American Thoracic Society, American College of Chest Physicians. ATS/ACCP statement on cardiopulmonary exercise testing. Am J Respir Crit Care Med. (2003) 167:211–77. doi: 10.1164/rccm.167.2.211, PMID: 12524257

[7] Kawachi S Fujimoto K. Metronome-paced incremental hyperventilation may predict exercise tolerance and Dyspnea as a surrogate for dynamic lung hyperinflation during exercise. Int J Chron Obstruct Pulmon Dis. (2020) 15:1061–9. doi: 10.2147/COPD.S246850, PMID: 32523336 PMC7237123

[8] O’Donnell DE. Dynamic lung hyperinflation and its clinical implication in COPD. Rev Mal Respir. (2008) 25:1305–18. doi: 10.1016/S0761-8425(08)75094-0, PMID: 19107020

[9] Guenette JA Webb KA O’Donnell DE. Does dynamic hyperinflation contribute to dyspnoea during exercise in patients with COPD? Eur Respir J. (2012) 40:322–9. doi: 10.1183/09031936.00157711, PMID: 22183485

[10] O’Donnell DE Laveneziana P. Physiology and consequences of lung hyperinflation in COPD. Eur Respir Rev. (2006) 15:61–7. doi: 10.1183/09059180.00010002

[11] Rossi A Aisanov Z Avdeev S Di Maria G Donner CF Izquierdo JL . Mechanisms, assessment and therapeutic implications of lung hyperinflation in COPD. Respir Med. (2015) 109:785–802. doi: 10.1016/j.rmed.2015.03.010, PMID: 25892293

[12] Klijnhout J Mannée D Van Den Heuvel M Van Den Borst B Van Helvoort H. Patients with chronic obstructive pulmonary disease can accurately perform home-based measurements of inspiratory capacity and dynamic hyperinflation. COPD: J Chron Obstruct Pulmon Dis. (2022) 19:236–42. doi: 10.1080/15412555.2022.2069554, PMID: 35535918

[13] Guenette JA Chin RC Cory JM Webb KA O’Donnell DE. Inspiratory capacity during exercise: measurement, analysis, and interpretation. Pulm Med. (2013) 2013:1–13. doi: 10.1155/2013/956081 PMC3582111 23476765

[14] Otto-Yáñez M Sarmento Da Nóbrega AJ Torres-Castro R Araújo PRS Carvalho De Farias CA Dornelas De Andrade ADF . Maximal voluntary ventilation should not be estimated from the forced expiratory volume in the first second in healthy people and COPD patients. Front Physiol. (2020) 11:537. doi: 10.3389/fphys.2020.00537, PMID: 32581835 PMC7296050

[15] Denolin H Sadoul P Orte N. L. L’Exploration fonctionnelle pulmonaire. Paris, France: Masson et Cie. (1964).

[16] Shephard RJ. Some factors affecting the open-circuit determination of maximum breathing capacity. J Physiol Lond. (1957) 135:98–113. doi: 10.1113/jphysiol.1957.sp005698, PMID: 13398969 PMC1358916

[17] D’Silva JL Mendel D. The maximum breathing capacity test. Thorax. (1950) 5:325–32. 14809667 10.1136/thx.5.4.325 PMC1018345

[18] Cara M Jouasset D. Maximum ventilation can be attained by a rebreathing experiment. CR Seances Soc Biol Fil. (1953) 147:1055–7. PMID: 13116586

[19] Johnson BD Weisman IM Zeballos RJ Beck KC. Emerging concepts in the evaluation of ventilatory limitation during exercise: the exercise tidal flow-volume loop. Chest. (1999) 116:488–503. doi: 10.1378/chest.116.2.488, PMID: 10453881

[20] Cooper CB Calligaro GL Quinn MM Eshaghian P Coskun F Abrazado M . Determinants of dynamic hyperinflation during metronome-paced tachypnea in COPD and normal subjects. Respir Physiol Neurobiol. (2014) 190:76–80. doi: 10.1016/j.resp.2013.08.002, PMID: 23994176

[21] Chuang ML Hsieh MJ Wu TC Lin IF. Developing a new marker of dynamic hyperinflation in patients with obstructive airway disease - an observational study. Sci Rep. (2019) 9:7514. doi: 10.1038/s41598-019-43893-1, PMID: 31101856 PMC6525207

[22] Ouattara S Siransy-Balayssac E Liliane Kondo A Augustin Yéo T Serges Dah C Bogui P. Gender influence on the MVV / FEVı ratio in a population of healthy young adults. Physiol Rep. (2020) 8:e14623. doi: 10.14814/phy2.14623, PMID: 33112050 PMC7592411

[23] Shah NM Kaltsakas G. Respiratory complications of obesity: from early changes to respiratory failure. Breathe. (2023) 19:220263. doi: 10.1183/20734735.0263-2022, PMID: 37378063 PMC10292783

[24] Balmain BN Halverson QM Tomlinson AR Edwards T Ganio MS Babb TG. Obesity blunts the ventilatory response to exercise in men and women. Ann Am Thorac Soc. (2021) 18:1167–74. doi: 10.1513/AnnalsATS.202006-746OC, PMID: 33465334 PMC8328370

